# Blood From Both Ears: A Hidden Mandibular Condyle Fracture Revealed by Otorrhagia

**DOI:** 10.7759/cureus.101808

**Published:** 2026-01-18

**Authors:** Akira Saito, Tatsuya Tanaka, Eiichi Suehiro, Akira Matsuno

**Affiliations:** 1 Department of Neurosurgery, International University of Health and Welfare, Narita Hospital, Narita, JPN

**Keywords:** external auditory canal fracture, hearing loss, mandibular condyle fracture, maxillofacial trauma, otorrhagia, otoscopic examination

## Abstract

Otorrhagia following mandibular trauma may appear straightforward; however, underlying external auditory canal (EAC) fractures and associated condylar injuries can be easily overlooked without targeted evaluation. We present the case of an 88-year-old man who developed bilateral ear bleeding and hearing loss after falling and striking his chin. Computed tomography revealed bilateral mandibular condyle fractures with associated fractures of the anterior walls of both EACs. The canals were filled with hematoma, and hearing loss was presumed to result from mechanical obstruction. Otoscopic examination after clot removal confirmed mucosal injury, and auditory symptoms improved following decompression and ear wick placement.

This case highlights a diagnostic pitfall in which clinicians may overlook condylar fractures as a cause of EAC injury. The mandibular condyle lies immediately inferior to the EAC anterior wall, and superior displacement during chin trauma may result in canal injury. Bilateral otorrhagia after chin trauma should therefore raise clinical suspicion for underlying mandibular condyle fractures and associated EAC disruption.

Radiological assessment with computed tomography (CT) and direct visualization via otoscopy are essential to detect these injuries early and guide conservative management. Our experience emphasizes that visible otorrhagia alone does not reveal the full extent of injury. Prompt clot removal and canal packing helped prevent adhesion and long-term auditory complications in our case.

Clinicians should maintain a high index of suspicion for EAC fractures and mandibular condyle injuries in patients with chin trauma and otorrhagia, even when bleeding is visibly apparent.

## Introduction

Mandibular condyle fractures are a common subtype of maxillofacial trauma, accounting for up to 56% of mandibular fractures [1]. These injuries often occur following blunt trauma to the chin, particularly in elderly individuals with reduced bone density and impaired protective reflexes [2].

Anatomically, the mandibular condyle articulates with the temporal bone at the temporomandibular joint (TMJ), which lies immediately inferior to the anterior wall of the external auditory canal (EAC). Posterior-superior displacement of the condyle during trauma can result in direct impaction against the EAC, potentially causing wall fracture, hematoma formation, or canal obstruction [3,4]. While this anatomical vulnerability is well understood, the actual clinical involvement of the EAC in mandibular trauma is frequently under-recognized, especially in the absence of overt otologic symptoms.

Lu et al. reported that 13.3% of mandibular condyle fractures were associated with EAC bleeding, and Jiang et al. noted that 44.7% of condylar head fractures and 35.6% of neck fractures involved the EAC anterior wall [3,4]. Despite these figures, otoscopic examination is not routinely performed in emergency settings, and radiologic evaluation may be limited to dental or mandibular views [3,5].

This report addresses this diagnostic gap by presenting a case of bilateral mandibular condyle fractures with bilateral anterior EAC wall fractures, leading to otorrhagia and transient hearing loss. The case highlights the importance of early otoscopic and computed tomography (CT) evaluation, as well as conservative EAC management to prevent long-term sequelae. This case contributes to the clinical understanding by providing detailed imaging, otoscopic findings, and audiometric follow-up that demonstrate the functional impact and recovery course of this overlooked injury pattern.

## Case presentation

An 88-year-old man fell while walking and struck his chin. Emergency services noted active bleeding from both ears. On arrival, he was alert and oriented, without focal neurological deficits. Physical examination revealed chin abrasions, bilateral cheek swelling, otorrhagia, bilateral hearing loss, and restricted mouth opening (interincisal distance: 20 mm). Vital signs were stable except for elevated blood pressure (197/108 mmHg).

**Figure 1:**
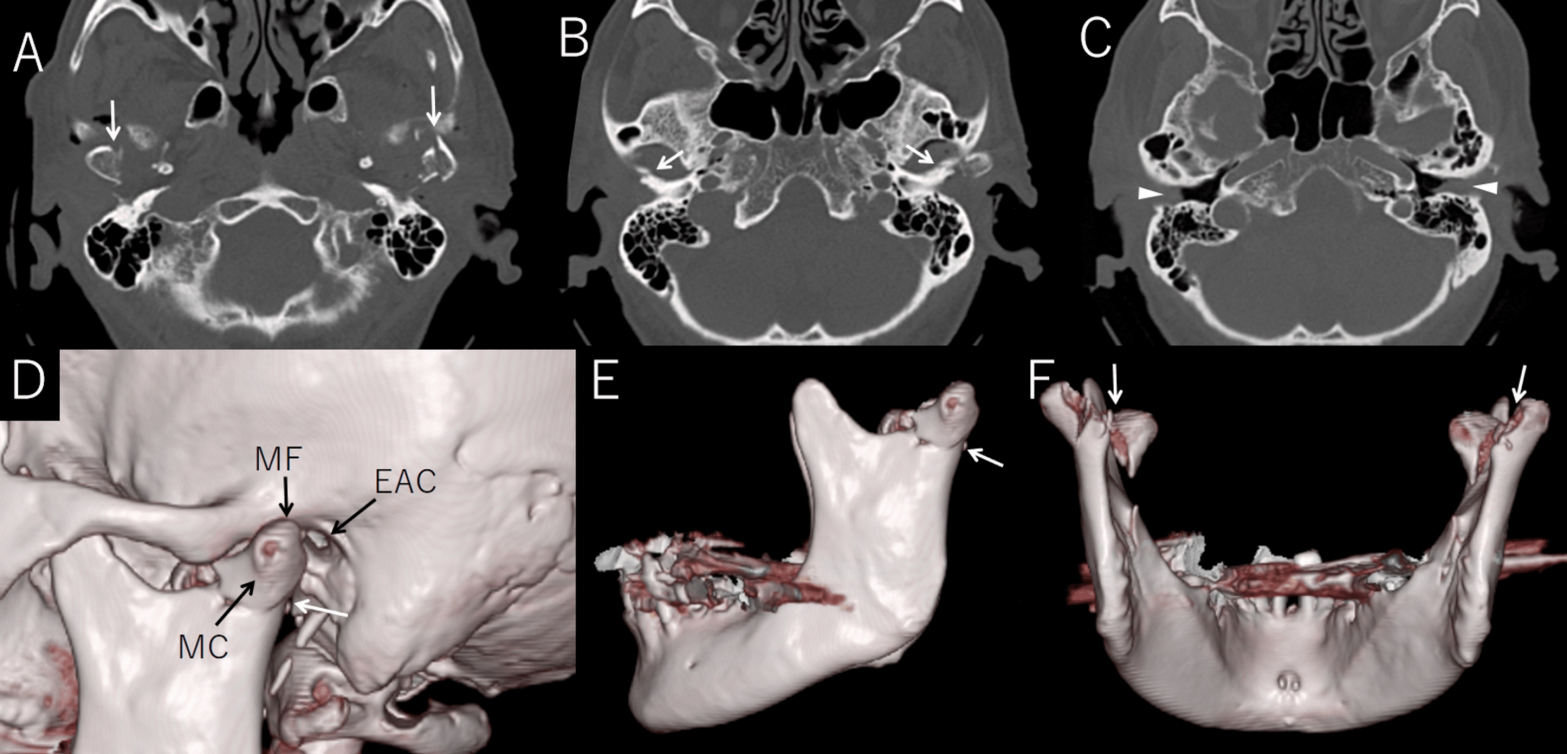
CT and 3D reconstruction images demonstrating bilateral mandibular condyle fractures and associated external auditory canal injury Axial CT images (A–C) show bilateral mandibular condyle fractures (white arrows in A), a fracture of the anterior wall of the left external auditory canal (white arrows in B), and soft tissue density in both external auditory canals indicating hematoma (arrowheads in C). Three-dimensional CT reconstructions (D–F) reveal the anatomical relationship between the mandibular condyle (MC), mandibular fossa (MF), and external auditory canal (EAC) (D), as well as bilateral condylar fractures clearly visible from lateral (E) and posterior (F) views (white arrows).

Head CT revealed bilateral fractures of the mandibular condyles with upward displacement of both condylar heads, but no dislocation from the glenoid fossa, classified as Spiessl and Schroll type II. Fractures of the anterior walls of both EACs were also noted, with hematoma filling the canals (Figure 1).

**Figure 2:**
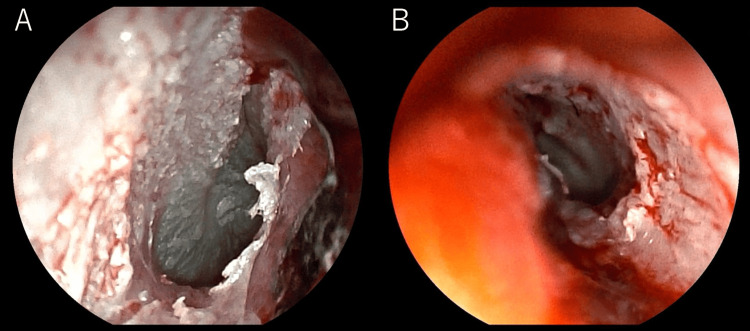
Otoscopic images of both ears after hematoma removal (A) Right external auditory canal. (B) Left external auditory canal. Both sides show edematous canal walls and mucosal injury with exposed tympanic membranes. These findings correlate with CT-demonstrated anterior external auditory canal wall fractures and otorrhagia.

Otoscopy showed swollen canals with dark red clots bilaterally. After gentle removal of the clots, the tympanic membranes were visualized and found to be intact, although mucosal injury and bleeding from the anterior EAC wall were present (Figure 2).

Gauze ear wicks were inserted to control bleeding and prevent canal adhesion. Pure tone audiometry performed after clot evacuation revealed mild bilateral sensorineural hearing loss: right 48.8 dB, left 46.3 dB. The hearing loss improved immediately following decompression.

Conservative treatment was initiated based on the absence of significant occlusal disturbance or dislocation. The patient was able to resume oral intake without difficulty.

## Discussion

In our case, bilateral mandibular condyle (MC) fractures accompanied by fractures of the anterior walls of the EACs led to hematoma-induced obstruction and transient hearing loss.

The MC articulates just inferior to the anterior wall of the EAC, forming the TMJ. In cases of facial trauma, superior displacement of the condyle can directly impact the anterior EAC wall, resulting in bony disruption, hematoma formation, and temporary conductive or sensorineural hearing loss [3-6]. In this patient, removal of obstructing clots led to the restoration of hearing, underscoring the importance of otoscopic evaluation in mandibular trauma cases with associated otologic symptoms.

Notably, EAC injuries secondary to condylar fractures are often under-recognized. A literature review by Lu et al. reported that 13.3% of MC fractures were associated with EAC bleeding [3]. Jiang et al. further found that EAC wall fractures occurred in 44.7% of condylar head (type I) fractures and 35.6% of condylar neck (type II) fractures [4]. Despite these substantial rates, otoscopic examination is not routinely performed in emergency settings unless patients present with overt otorrhagia or hearing complaints.

Post-traumatic EAC stenosis is a recognized complication, particularly when hematomas are not properly evacuated [7,8]. In our case, the insertion of ear wicks (gauze packing) helped prevent canal adhesions and supported mucosal healing, aligning with existing recommendations for EAC fracture management. As noted in previous reports, failure to maintain canal patency may result in fibrosis and long-term conductive hearing loss [3,4,6].

This case reinforces the clinical message that otorrhagia following mandibular trauma warrants careful evaluation for possible EAC involvement, especially in the presence of condylar fractures. Given the anatomical proximity and shared mechanisms of injury, both CT imaging and otoscopic assessment are essential for timely diagnosis and conservative management planning.

Moreover, this case is particularly notable due to its bilateral presentation in an elderly patient. While prior studies have primarily focused on unilateral injuries, bilateral cases remain uncommon and underreported [3,4,6]. Age-related bone demineralization and decreased tissue elasticity may predispose older adults to bilateral condylar fractures, even after relatively low-energy trauma such as a simple fall [2]. This highlights the importance of considering skeletal fragility in geriatric facial trauma.

## Conclusions

This case highlights an important yet often under-recognized injury mechanism in mandibular trauma. Because of the close anatomical proximity of the MC to the EAC, condylar fractures may be accompanied by EAC injury, particularly in patients presenting with otorrhagia following chin trauma. Although the clinical course in this case was favorable, the primary purpose of this report is educational: to increase awareness of this association and emphasize the importance of comprehensive evaluation, including both otoscopic and radiologic assessments, when managing mandibular trauma with otologic symptoms.

## References

[1] Kozakiewicz M, Walczyk A (2023). Current frequency of mandibular condylar process fractures. J Clin Med.

[2] Nogami S, Yamauchi K, Yamashita T, Kataoka Y, Hirayama B, Tanaka K, Takahashi T (2015). Elderly patients with maxillofacial trauma: study of mandibular condyle fractures. Dent Traumatol.

[3] Lu C, He D, Yang C (2014). Which craniofacial fractures are associated with external auditory canal bleeding?. J Oral Maxillofac Surg.

[4] Jiang Y, Jiang C, Huang X (2022). Associations between condylar fractures and external auditory canal fracture: a 7-year retrospective study. J Craniomaxillofac Surg.

[5] Panneerselvam E, Alagesan RC, Sripathi V, Sridharan G, Balasubramanian S, Balakrishna KR (2023). External auditory canal injuries in maxillofacial trauma - proposal of a symptom-based treatment algorithm with a report of twelve cases. Natl J Maxillofac Surg.

[6] Dang D (2007). Bilateral mandibular condylar fractures with associated external auditory canal fractures and otorrhagia. Radiol Case Rep.

[7] Burchhardt DM, David J, Eckert R, Robinette NL, Carron MA, Zuliani GF (2015). Trauma patterns, symptoms, and complications associated with external auditory canal fractures. Laryngoscope.

[8] Loh FC, Tan KB, Tan KK (1991). Auditory canal haemorrhage following mandibular condylar fracture. Br J Oral Maxillofac Surg.

